# Effects of the establishment of trauma centres on the mortality rate among seriously injured patients: a propensity score matching retrospective study

**DOI:** 10.1186/s12873-023-00776-z

**Published:** 2023-01-19

**Authors:** Qiangping Zhou, Haijin Huang, Linhui Zheng, Haiming Chen, Yuanlin Zeng

**Affiliations:** 1 Department of Emergency Surgery, The First Affiliated Hospital of Nanchang University, Nanchang, 17 Yongwaizheng Street, Nanchang, 330006 Jiangxi China; 2 Department of Anesthesiology, The First Affiliated Hospital of Nanchang University, Nanchang, China

**Keywords:** Trauma centres, Mortality, Injury severity score, Retrospective studies

## Abstract

**Background:**

Little evidence suggests that trauma centres are associated with a lower risk of mortality in severely injured patients (Injury Severity Score (ISS) ≥16) with multiple injuries in China. The objective of this study was to determine the association between the establishment of trauma centres and mortality among severely injured patients with multiple injuries and to identify some risk factors associated with mortality.

**Methods:**

A retrospective single-centre study was performed including trauma patients admitted to the First Affiliated Hospital of Nanchang University (FAHNU) between January 2016 and December 2021. To determine whether the establishment of a trauma centre was an independent predictor of mortality, logistic regression analysis and propensity score matching (PSM) were performed.

**Results:**

Among 431 trauma patients, 172 were enrolled before the trauma centre was built, while 259 were included after the trauma centre was built. A higher frequency of older age and traffic accident injury was found in patients diagnosed after the trauma centre was built. The times for the completion of CT examinations, emergency operations and blood transfusions in the “after trauma centre” group were shorter than those in the “before trauma centre” group. However, the total expenditure of patients was increased. In the overall group, univariate and multivariate logistic regression analyses showed that a higher ISS was an independent predictor for worse mortality (OR = 17.859, 95% CI, 8.207–38.86, *P* < 0.001), while the establishment of a trauma centre was favourable for patient survival (OR = 0.492), which was also demonstrated by PSM. After determining the cut-off value of time for the completion of CT examination, emergency operation and blood transfusion, we found that the values were within the “golden one hour”, and it was better for patients when the time was less than the cut-off value.

**Conclusion:**

Our study showed that for severely injured patients, the establishment of a trauma centre was favourable for a lower mortality rate. Furthermore, the completion of a CT examination, emergency surgery and blood transfusion in a timely manner and a lower ISS were associated with a decreased mortality rate.

## Introduction

Injury caused by trauma is a leading cause of mortality, disability, financial expenditure, and health service utilization in China, especially for youth, and is considered a life-threatening “modern disease” [1]. According to statistics, traumatic injury causes more than 700 thousand deaths each year, accounting for 9% of all deaths and ranking as the fifth leading cause of death [2]. Among the mechanisms, with the development of society, traffic accidents have become the main cause of injury; the overall mortality rate of traffic accidents could be up to 33.87%, especially in China and other countries [3, 4].

In recent years, with the introduction of trauma centres in many countries, great improvement has achieved in the outcomes of injured patients; for instance, a recent meta-analysis proved that an efficient trauma centre could decrease the mortality rate of patients by 15% [5, 6]. However, some studies reported that in well-organized trauma centres, the rate of medical errors could be up to 20%; the rates were significantly higher in these centres than in same-level trauma hospitals [7–9]. Therefore, many models to evaluate trauma centre performance have been proposed, which cover mortality, costs, adverse events, resource use and quality of life [10, 11]. Evelyn et al. proposed the question of whether an increased number of trauma centres was better for injury-related mortality. After performing some research, they concluded that more centres did not improve injury-related mortality [12, 13]; therefore, more work is needed to identify the type and number of trauma centres needed to improve injury-related mortality. Interdisciplinary cooperation and the provision of general surgical therapy in trauma centres are urgent and necessary; however, the picture of trauma centres is complicated, and evidence to support the establishment of trauma centres remains insufficient [14–16]. Moreover, in China, the establishment of trauma centres is in the initial stage [1]. Hence, the effect of trauma centres, as an integrated care system that is large and complex, should be explored by focusing on performance comparisons between trauma centres and non-trauma centres.

To monitor and improve the quality of trauma centres, we performed a retrospective study by enrolling severely injured patients with multiple injuries who were diagnosed before and after the establishment of a trauma centre from 2016 through 2021. Performance evaluation could enable clinicians to identify and address problems associated with the trauma care system and how to best use it.

## Methods

### Patient selection

**Fig. 1:**
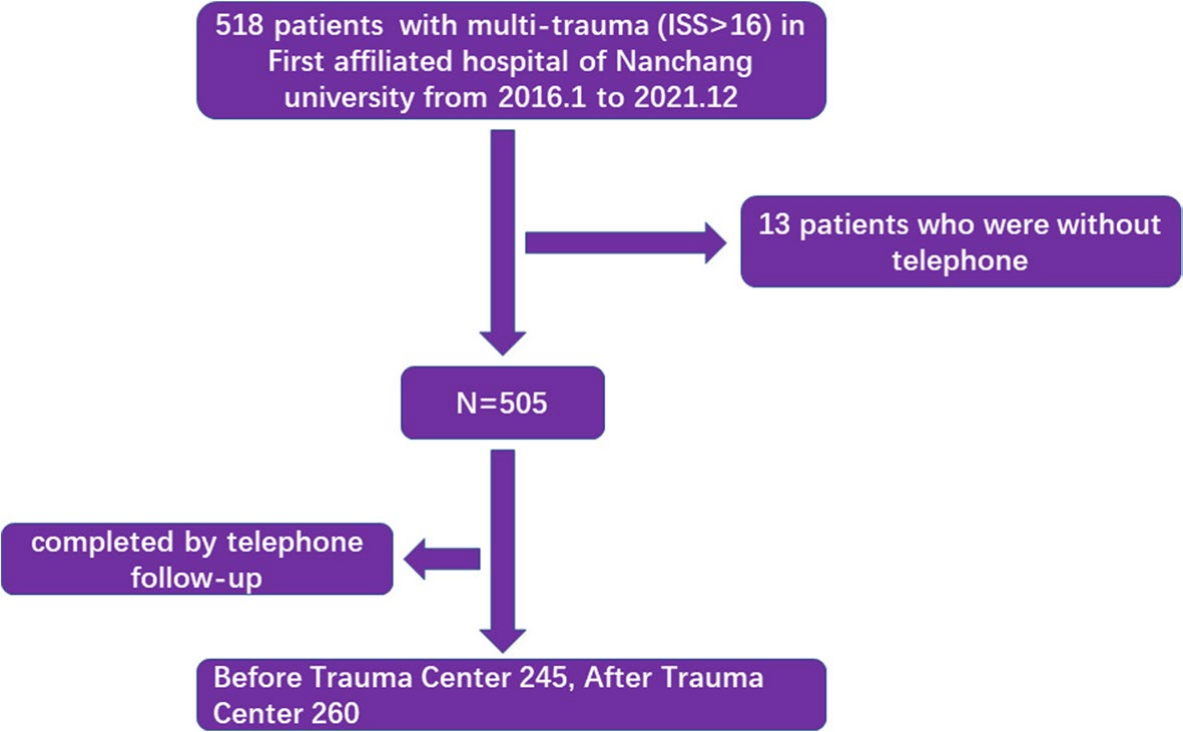
Flow diagram of our study. A total of 431 patients were enrolled and divided into either the “before trauma centre” group (*n* = 172, admitted from January 2016 to December 2018) or the “after trauma centre” group (*n* = 259, admitted from January 2019 to December 2021)

All patients were selected from the First Affiliated Hospital of Nanchang University (FAHNU). In the selection of patients from our centre, we chose patients who were admitted from January 2016 through December 2021 to collect clinical characteristic data. The inclusion criteria were as follows: (1) met the diagnostic criteria of an injury severity score (ISS) ≥ 16, and had multiple injuries involving at least 2 or more parts of the body, including cranial injury (skull fracture, intracranial haematoma with coma, and so on), neck injury (such as a cervical spine injury), thoracic injury (multiple rib fractures, diaphragmatic hernia, etc.), abdominal injury (intraperitoneal haemorrhage, etc.), damage to the urogenital system, pelvic fracture, spinal fracture and limb injuries; and (2) the time from trauma to admission was less than 24 hours. The exclusion criteria were as follows: (1) an ISS < 16; (2) the injury only included a single part of the body; (3) serious basic diseases, such as coagulation dysfunction, severe cardiopulmonary disease, severe hypertension, severe liver and kidney function damage, and severe hypoproteinaemia. Accordingly, 172 patients admitted from January 1, 2016, to December 30, 2018, were enrolled into a “before trauma centre” group and 259 patients admitted from January 1, 2019, to December 30, 2021, were included in an “after trauma centre” group. All our patients were followed up by telephone, and those who were lost to follow-up were excluded. Informed consent was obtained from all subjects and/or their legal guardian(s). The characteristic information of patients from the FAHNU is provided in Table 1, while a flowchart of the included patients is shown in Fig. 1.

**Table 1:** Basic information of patients with trauma from FAHNU diagnosed from 2016 through 2021

Variables	Total	Before trauma center	After trauma center	***P*** value
Total	431	172	259	
**Gender**				0.186
Male	329 (74.65%)	137 (79.6%)	192 (74.13%)	
Female	102 (25.34%)	35 (20.35%)	67 (25.87%)	
**Age**				*0.008*
< 65	344 (79.81%)	148 (86.05%)	196 (75.68%)	
> =65	87 (20.19%)	24 (13.95%)	63 (24.32%)	
**Mechanism of injury**				*0.012*
Traffic accident	206 (47.8%)	71 (41.27%)	135 (52.12%)	
Fall	101 (23.43%)	38 (22.09%)	63 (24.32%)	
Other causes	124 (28.77%)	63 (36.64%)	61 (23.55%)	
**ISS score (median, IQR)**	21 (17–75)	22 (19–50)	21 (17–75)	*0.019*
**Injury Severity Score category**				0.896
16–24	264 (61.25%)	106 (61.63%)	158 (61%)	
> =25	167 (38.75%)	66 (38.37%)	101 (39%)	
**Time for completion of CT examination**				
**Yes (min)**	39 (30–720)	43 (30–720)	35 (26–180)	*< 0.001*
**No**	30 (6.96%)	9 (5.23%)	21 (8.11%)	
**Surgery**				0.5409
No	159 (36.89%)	60 (34.88%)	99 (38.22%)	
Yes	272 (63.11%)	112 (65.12%)	160 (61.78%)	
Selective surgery	63	37	26	
Emergency (min)	62 (53–566)	120 (84–473)	57.5 (50–566)	*< 0.001*
**blood transfusion**				
No	292 (67.75%)	127 (73.84%)	165 (63.71%)	
Yes (min)	46 (38–183)	105 (40–183)	42 (38–70)	< 0.001
**Length of stay in ICU**	2 (0–98)	0 (1–98)	2 (0–67)	0.735
**Length of stay in hospital**	16 (9–98)	15 (8–98)	17 (9–89)	0.334
**Total expenses**	91,727 (39,109.55–850,955.2)	91,727 (39130–850,955)	97,728 (49542–850,955.2)	*0.005*
**Outcome**				*0.042*
Non deaths	291 (83.53%)	136 (79.07%)	224 (86.49%)	
deaths	71 (16.47%)	36 (20.93%)	35 (13.51%)	

Italic values indicate statistical significance when *P* < 0.05

### Definitions of variables

In this study, the clinical features extracted from the FAHNU included age, sex, mechanism of injury, ISS, time to completion of a CT examination, surgery, and blood transfusion, length of stay in the ICU, total expenses, length of stay in the hospital and outcome. Sex was recorded as male or female. Age was separated into < 65 and ≥ 65 years. Mechanisms of injury included traffic accidents, falls and other causes, which included penetrating wounds and blast injuries [17]. The time for the completion of a CT examination was recorded as the actual value. Surgery status was recorded as No or Yes, and was separated into emergency operations and selective surgeries. Blood transfusion status, length of stay in the ICU, total expenses, and length of stay in the hospital were recorded as the actual values. Some definitions should be explained: (1) the time to completion of a CT examination indicates the time from the registration of the patient to the completion of a CT examination. (2) The time to blood transfusion (blood transfusion preparation time): for patients who needed a blood transfusion, the time from the issuance of the blood transfusion application form to the start of the blood transfusion was recorded, including the following: when the nurse drew blood for blood preparation; when the laboratory determined the patient’s blood type; when the blood transfusion department conducted the blood cross, blood matching, and blood distribution and delivered the blood to the rescue room; and when the nurse checked the patient’s blood type and the blood transfusion was started. The time to blood transfusion preparation was compared in this article, reflecting the timeliness of establishing a trauma centre to deal with critical patients, so is the results are comparable. (3) The time to the start of surgery referred to the time from the visit to the start of the surgery in the operating room for patients who required surgery, including the time of the preoperative conversation and signature, anaesthesia preparation and anaesthesia operation, rather than the time from the visit to the completion of surgery. Because different trauma patients require different hand surgery methods, it is meaningless to compare the time from the visit to the completion of the surgery. What we aimed to reflect in this article is the timeliness of the treatment of critical patients with multiple injuries, so we could only compare the time from seeing a doctor to the beginning of surgery. The main observation feature was the outcome of patients, which included recovery and death.

### Statistical analysis

For basic analysis, all included patients were divided into the “before trauma centre” group and the “after trauma centre” group according to the setting of the trauma centre; then, the associated clinical characteristics were compared via Pearson’s chi-squared test and the nonparametric Kruskal–Wallis rank-sum test. Univariate and multivariate logistic regression analyses were used to investigate the potential risk factors associated with mortality due to trauma, and the results are shown by odds ratios (ORs) with 95% confidence intervals (CIs). Regarding the imbalance of basic information, we performed propensity score matching (PSM) to obtain new balanced data for analysis when the *P* value was more than 0.05; the calliper value was set as 0.02. All statistical analyses were performed in R software, and all associated packages were obtained from the R software program website (https://cran.r-project.org/web/packages/). Student’s t test was used for continuous variables with a Gaussian distribution, and the nonparametric Kruskal–Wallis rank-sum test was used for nonnormally distributed continuous variables or ordinal categorical variables.

## Results

### Basic analysis of the included patients’ characteristics

In our study, as shown in Fig. 1, we enrolled a total of 431 patients, including 172 patients diagnosed before the trauma centre was built and 259 patients diagnosed after the trauma centre was built. All patient information is listed in Table 1. Comparing the two groups, the distribution of sex was similar (*P* = 0.186), while the age of the patients diagnosed after the trauma centre was built was older than that of patients before the trauma centre was built (≥65, 24.32% vs. 13.95%, *P* = 0.008). Regarding the mechanism of injury, the ratio of traffic accident injuries was significantly increased in the “after trauma centre” group (52.12% vs. 41.27%, *P* = 0.012). The ISS between the two groups was different (*P* = 0.019). The distribution of ISS category [18] was not significantly different, nor was the surgery selected. However, the time to an emergency operation in the “after trauma centre” group was markedly shorter than that in the “before trauma centre” group (*P* < 0.001). In line with this, the time to completion of a CT examination in patients diagnosed after the trauma centre was built was markedly decreased (*P* < 0.001), as well as the time to blood transfusion. The length of stay in the hospital and in the ICU did not vary with the setting of the trauma centre (*P* > 0.05). The total expenses of patients diagnosed after the trauma centre was built were higher than those of patients diagnosed before the trauma centre was built (*P* = 0.005). In the “before trauma centre” group, the mortality rate was 20.93%, which was significantly higher than that (13.51%) in the “after trauma centre” group (*P* = 0.042).

### Identifying risk factors for mortality in trauma patients and determining that the establishment of a trauma Centre was a favourable factor for lowering the mortality rate

**Fig. 2:**
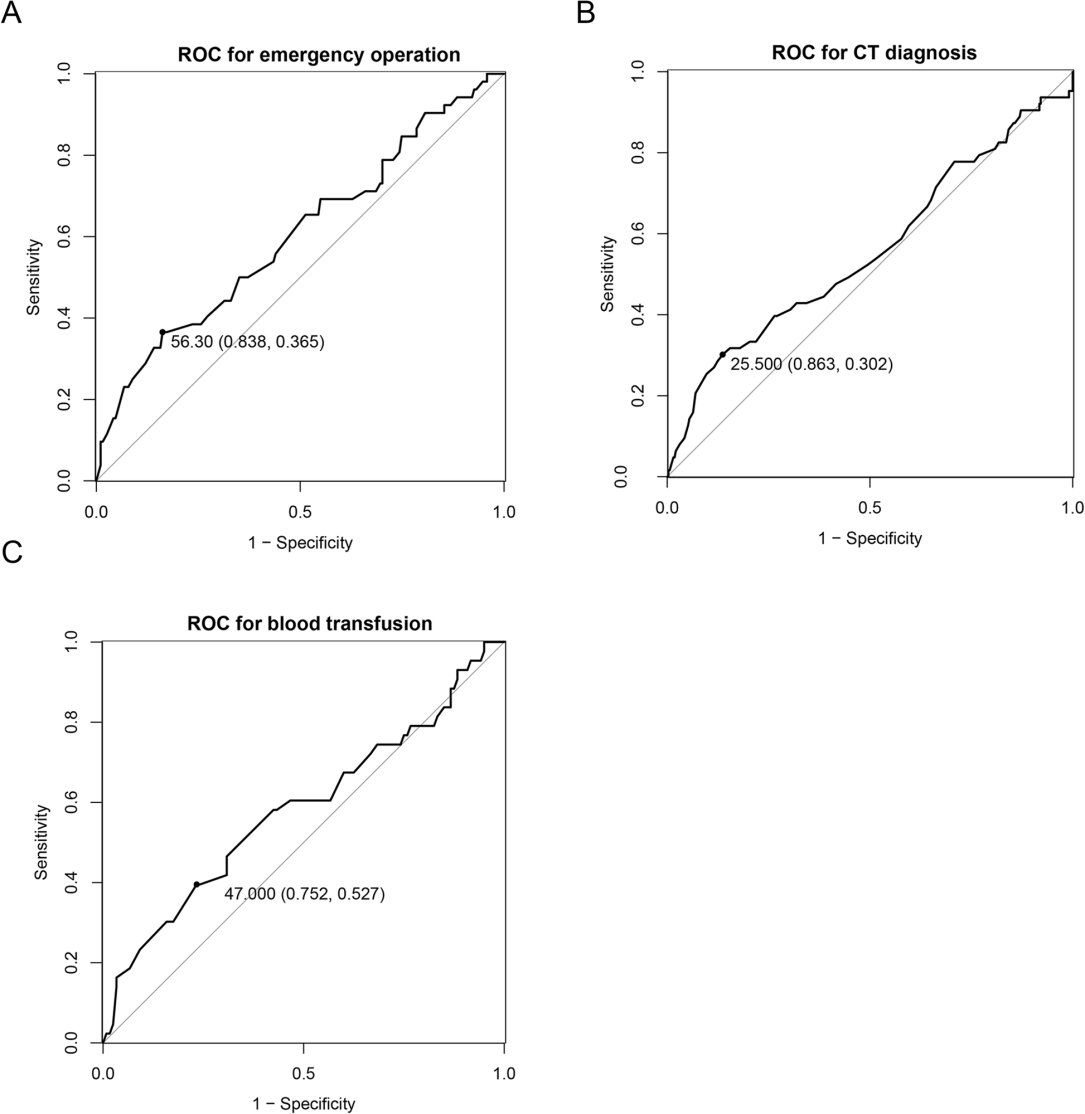
ROC curves of the time to completion of an emergency operation (**A**), a CT examination (**B**) and a blood transfusion (**C**) for predicting mortality. The cut-off value is marked in the ROC curves

To investigate the risk factors for mortality in trauma patients, we performed univariate and multivariate logistic regression analyses (Table 2). Patients with severe injuries and significantly high ISSs (ISS ≥25) also had a higher risk of mortality than those who had an ISS of 16–24 (OR, 14.617, *P* < 0.001). Admission to the ICU was a risk factor for mortality (*P* < 0.05), while the establishment of a trauma centre was a protective factor for mortality (OR, 0.59, 95% CI, 0.354–0.985, *P* = 0.04). To decrease the effect of confounding factors, we further performed multivariate logistic analysis. In the multivariate model, we found that a higher ISS remained a risk factor for mortality. The trauma centre setting was a favourable factor for a lower rate of mortality (OR, 0.492, *P* = 0.017); however, admission to the ICU was not an independent factor for mortality. Regarding the time to completion of a CT examination, an emergency operation and a blood transfusion were continuous variables, and they were associated with mortality from injury. We generated ROC curves to identify the cut-off values. As shown in Fig. 2, the cut-off values of time to completion of a CT examination, an emergency operation and a blood transfusion were 25.5, 56.3 and 47 min, respectively. Hence, according to the cut-off value, we divided patients into two groups, which are shown in Table 3. Based on the results of univariate analysis, we found that a time of more than 25.5 min to completion of a CT examination was a risk factor for patient mortality (OR, 2.725, 95% CI, 1.461–5.082, *P* = 0.002). Similarly, we found that a time to emergency surgery within 56.3 min was protective for patient survival (OR, 2.692, *P* = 0.002). Additionally, the completion of a blood transfusion should be occur within 47 min (OR, 1.86, *P* = 0.048). Considering that basic information, such as age and ISS, was not balanced, we performed PSM to adjust for these factors. As shown in Table 4, the distribution difference of basic characteristics was balanced because all *P* values were more than 0.05. The chi-squared test of the outcome variable showed that patients diagnosed after the establishment of a trauma centre had a lower rate of mortality (11.01% vs. 22.02%, *P* = 0.028). Furthermore, after balancing, the time to completion of a CT examination, an emergency operation and a blood transfusion remained shorter in the “after trauma centre” group, suggesting that the establishment of a trauma centre could shorten this time.

**Table 2:** Univariate and multivariate logistic regression model for exploring the potential risk factors for patients’ mortality

Variables	Univariate analysis	***P*** value	Multivariate analysis	***P*** value
**Gender**				
Male	Reference	–		
Female	1.317 (0.802–2.011)	0.393		
**Age**				
< 65	Reference	–		
> =65	1.304 (0.713–2.387)	0.389		
**Mechanism of injury**				
Traffic accident	Reference	–		
Fall	1.24 (0.680–2.259)	0.483		
Other causes	0.601 (0.310–1.165)	0.132		
**Injury Severity Score category**				
16–24	Reference	–	Reference	–
> =25	14.617 (7.215–29.613)	*< 0.001*	17.859 (8.207–38.86)	*< 0.001*
**Surgery**				
No	Reference	–		
Yes	0.712 (0.425–1.193)	0.197		
**blood transfusion**				
No	Reference	–		
Yes	1.569 (0.93–2.649)	0.092		
**Stay in ICU**				
No	Reference	–	Reference	–
Yes	1.972 (1.129–3.444)	*0.017*	0.682 (0.344–1.349)	0.271
**Trauma center**				
No	Reference	–	Reference	–
Yes	0.59 (0.354–0.985)	*0.04*	0.492 (0.275–0.879)	*0.017*

Italic values indicate statistical significance when *P < 0.05*

**Table 3:** Univariate logistic regression analysis for exploring factors for patients’ mortality

Variables	Univariate analysis	***P*** value
**Time for completion of CT examination (min)**		
< 25.5	Reference	–
> =25.5	2.725 (1.461–5.082)	0.002
**Emergency Surgery (min)**		
< 50.5	Reference	–
> =50.5	2.692 (1.201–4.869)	0.002
**blood transfusion (min)**		
< 47	Reference	–
> =47	1.86 (1.022–3.518)	0.048

**Table 4:** Basic information of patients from FAHNU after propensity-score-matching

Variables	Total	Before trauma center	After trauma center	***P*** value
Total	218	109	109	
**Gender**				0.435
Male	163 (74.77%)	84 (77.06%)	79 (72.48%)	
Female	55 (25.23%)	25 (22.94%)	30 (27.52%)	
**Age**				0.206
< 50	181 (83.03%)	94 (86.24%)	87 (79.82%)	
> =50	37 (16.97%)	15 (13.76%)	22 (20.18%)	
**Mechanism of injury**				0.222
Traffic accident	100 (45.87%)	56 (51.38%)	44 (40.37%)	
Fall	57 (26.15%)	24 (22.01%)	33 (30.28%)	
Other causes	61 (27.98%)	29 (26.61%)	32 (29.35%)	
**ISS score (median, IQR)**	21 (17–75)	21 (17–59)	21 (17–59)	0.714
**Time for completion of CT examination**				
**Yes (min)**	38 (29–720)	44.5 (30–720)	36.5 (25–68)	< 0.001
**Surgery**				0.889
No	83 (41.21%)	42 (38.53%)	41 (37.61%)	
Yes	135 (58.79%)	67 (61.47%)	68 (62.39%)	
Selective surgery		24 (22.01%)	7 (6.42%)	
Emergency (min)	67 (52–566)	63 (52–566)	58 (47.2–566)	< 0.001
**blood transfusion**				
No	160 (73.39%)	90 (78.9%)	70 (64.22%)	*0.016*
Yes (min)	46 (38–85)	105 (69–175)	42 (38–62)	< 0.001
**Outcome**				*0.028*
Recovery	182 (83.49%)	85 (77.98%)	97 (88.99%)	
death	36 (16.51%)	24 (22.02%)	12 (11.01%)	

Italic values indicate statistical significance when *P* < 0.05

## Discussion

In our study, we evaluated the effect of the establishment of a trauma centre on in-hospital mortality among injured patients. Our study suggests that trauma centres can provide definitive care for severely injured patients quickly and effectively as patients are centralized. Our findings are in line with those of previous studies, in which there is a relationship between better outcomes and the centralization of severely injured patients. Moreover, this study showed a very interesting result: we identified the best cut-off value for rescue time, including the time to completion of CT examinations and blood transfusions.

China is the largest developing country in the world and has a unique background [6]. With social development, the mechanism of trauma injury has greatly changed; for example, the ratio of injury induced by traffic accidents has significantly increased (Table 1). Before the establishment of a trauma centre, our trauma rescue team wasted much time before admission to the hospital and had no effective treatment for injured patients, leading to a 30% mortality rate [19]. However, we found that the value decreased to 13.51% after the trauma centre was built, of which the variance was significantly different. A previous study also found that the mortality rate of severe trauma patients was reduced from 33.82 to 20.49% [20, 21]. After the establishment of the trauma centre, the emergency multiple discipline team (EMDT) was launched for treatment in the trauma hospital [22, 23]. Compared with that of the traditional mode of rescue, the efficiency of rescue by the EMDT is greatly improved, which is why the time to completion of a CT examination, an emergency operation and a blood transfusion are significantly shortened [23, 24].

In our study, we identified a higher ISS as an independent risk factor for mortality in injured patients, which was in accordance with previous studies [25, 26]. Currently, the severity of trauma is based on the ISS system, and severe trauma is considered when the ISS is above 15 [27]. Regarding the effect of sex on mortality, some studies have confirmed that female traumatic injury patients have an obvious survival advantage compared to male patients; however, several articles also revealed that females had a lower overall mortality [28–30]. Consistent with our study, other studies have failed to show an association between sex and mortality [30, 31]. Before and after PSM, we found that the mortality of patients after the trauma centre was built decreased from 13.51 to 11.01%, while the mortality of patients before the trauma centre was built increased, suggesting that there were some confounding factors that decreased the effect of trauma centre establishment. Indeed, trauma centres improve the outcomes of injured patients, and a developed trauma centre helps decrease the mortality rate of trauma by 13% [32, 33].

It is well known that severe trauma rescue is time-dependent, and the concept of the “golden one hour” is proposed to emphasize the importance of time in trauma rescue [34, 35], which was also demonstrated by our study. Based on this result, the treatment of severe trauma patients should be performed within 1 h, which is considered a key determinant of the outcomes of these patients [17, 36]. Furthermore, the univariate logistic analysis also demonstrated that the longer the time to therapy was, the worse the outcome (Table 3). In line with our study, a previous study also found that the treatment time was reduced after the establishment of a trauma centre [19, 20]. In China, government departments and medical institutions have realized the importance of severe trauma centre construction and established the national trauma care centre in 2018 [37–39]. Hence, to our knowledge, our hospital, the FAHNU, was organized to set up a medical centre in the field of trauma with the help of the national trauma centre. Our retrospective study is the first to demonstrate that trauma centres reduce the mortality and disability rates of trauma patients. However, the in-hospital cost was obviously increased after the trauma centre was built. A few reasons could explain this result. First, the length of stay in the ICU was increased due to effective rescue by the trauma centre, resulting in increased expenditure. Second, with the development of the economy and the improvement of medical resources, our hospital introduced much advanced equipment, such as extracorporeal membrane oxygenation (ECMO), leading to increased in-hospital costs.

Our study has some limitations that should be noted. First, our study was a retrospective study from a single centre, resulting in a limited number of cases. Second, we could not compare the outcomes of patients with severe injuries between young and older patients because there was a difference in age distribution. Usually, there is no difference in medical care procedures in patients of different ages [40]. Furthermore, physicians and other clinical workers had different levels of experience before and after the trauma centre was built, which was associated with patient mortality and was not evaluated in our study. Additionally, data on the time to transfer and other treatments were lacking in our study. Therefore, in the future, we intend to conduct a more profound and detailed survey to verify the efficacy of the trauma centre.

In conclusion, the trauma centre decreased the mortality of severely injured patients. In addition, the time to completion of a CT examination, an emergency operation and a blood transfusion should be within 25.5, 56.3 and 47 min, respectively. Our results provide evidence to support that trauma centres are favourable for patient mortality and advise clinicians to pay more attention to building trauma centres. Furthermore, our study also supports that the time from hospital admission to the completion of a CT examination and other definitive care measures should be within 1 h. In the future, we can efficiently reduce the mortality rate of severely injured patients by transferring patients from a larger area into the corresponding trauma centre.

## Data Availability

The datasets used and/or analyzed during the current study available from the corresponding author on reasonable request.
